# Distal ureteral stone formation over migrated Hem-o-lok clip after robot-assisted partial nephrectomy

**DOI:** 10.1016/j.ijscr.2019.03.024

**Published:** 2019-03-30

**Authors:** Murat Can Kiremit, Ersin Koseoglu, Omer Acar, Mert Kilic, Yakup Kordan, Abdullah Erdem Canda, Mevlana Derya Balbay, Tarık Esen

**Affiliations:** a Department of Urology, Koc University Hospital, Istanbul, Turkey; b Department of Urology, Koc University, School of Medicine, Istanbul, Turkey; c Department of Urology, VKF Amerikan Hospital, Istanbul, Turkey

**Keywords:** Clips, Minimally invasive surgery, Urinary lithiasis, Case reports

## Abstract

**Introduction:**

Hem-o-lok clip migration into the ipsilateral collecting system and formation of a distal ureteral stone after robot-assited partial nephrectomy (PN) is a rare condition of stone disease.

**Presentation of case:**

A 48-year-old male presented with hematuria. Physical examination was unremarkable. Urinalysis showed presence of red blood cells. Serum multiple analysis and ultrasonography were within normal limits. Contrast enhanced abdominal computerized tomography scan revealed an 8-mm right distal ureteral stone, which was not associated with ipsilateral hydronephrosis.

**Discussion:**

With the widespread adoption of minimally invasive surgery, it is not uncommon to utilize foreign bodies as surgical facilitators which also have the potential to migrate to the urothelium-lined urinary tract and act as a stone nidus when used for endoscopic PN

**Conclusion:**

Clinicians should be aware of the possibility of migrated hem-o-lok clips serving as a nidus for urinary tract stone formation in patients who have undergone endoscopic PN.

## Highlights

• Foreign bodies, such as suture materials, mesh, cotton swab, Hem-o-lok clips, metallic clips, coils used for angioembolization etc. are rare causes of urinary tract stone disease.
• Clinicians should be aware of the possibility of migrated hem-o-lok clips serving as a nidus for urinary tract stone formation in patients who have undergone endoscopic PN.
• Attention to suture tension during endoscopic partial nephrectomy may reduce the risk of clip migration.
• Laser lithotripsy of the calculous cortical rim around the Hem-o-lok clip(s) and removal of the denuded foreign body under direct endoscopic visualization are strongly advisable, since Hem-o-lok clips are usually SWL-resistant.

## 1 Introduction

Owing to the increased utility of cross-sectional abdominal imaging studies, the number of incidentally detected renal masses has increased substantially. These tumors are usually small and amenable to excision by partial nephrectomy (PN) [1]. In the era of minimally invasive surgery, pure laparoscopic or robot-assisted laparoscopic surgery (endoscopic surgery) have become increasingly popular for the treatment of such renal masses [2].

In order to decrease the risk of renal functional deterioration, every effort is being employed to minimize the warm-ischemia time (WIT) during PN. Renorrhaphy can be regarded as one of the most technically challenging steps of endoscopic PN and it has a definite impact on WIT [3]. Hem-o-lok clips (Weck, Teleflex Medical, Research Triangle Park, NC, USA), which are used to secure sutures instead of conventional knot tying, have been introduced to expedite parenchymal reconstruction during endoscopic PN [4].

Herein, we report a patient who was diagnosed with a distal ureteral stone after robot-assisted PN. One of the Hem-o-lok clips that were applied during robot-assisted PN had eroded into the collecting system, migrated to distal ureter and acted as a nidus for stone formation. The paper has been reported in line with the SCARE criteria [5]

## 2 Case presentation

**Fig. 1:**
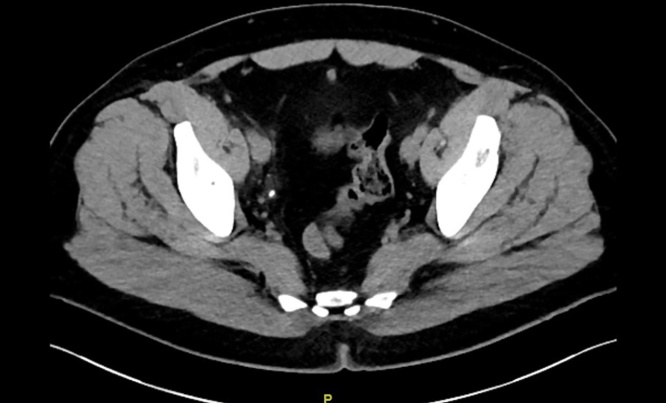
Abdominal computerized tomography (CT) scan revealed an 8-mm right distal ureteral stone.

A 48-year-old male presented with gross hematuria. Physical examination was unremarkable. Microscopic examination of the urine sediment revealed abundance of red blood cells. Serum analyses and ultrasonographic evaluation of the urinary tract were unremarkable. Contrast-enhanced abdominal computerized tomography (CT) scan demonstrated an 8-mm right distal ureteral stone, without ipsilateral hydronephrosis (Fig. 1). An initial trial of medical expulsive therapy was declared to have failed after 6 weeks. Based on CT attenuation of the calculus (1609 Hounsfield Units - HU), ureteroscopic surgery was favored over shock-wave lithotripsy (SWL).

Prior medical and surgical history including urinary system stone disease, were unremarkable other than a robot-assisted PN that performed two years ago for an incidentally detected renal mass. When the operative report and visual recordings were reviewed it was understood that after removing the renal mass, renorrhaphy had been performed in a single-layered fashion using a running suture. Renal parenchyma was compressed using Hem-o-lok clips at the renal capsule [6]. He had received blood transfusions in the early postoperative period due to clinically significant hemoglobin decline. Pathological examination revealed pT1a, Fuhrman grade 2, clear cell RCC with negative surgical margins.

### 2.1 Ureteroscopic intervention

**Fig. 2:**
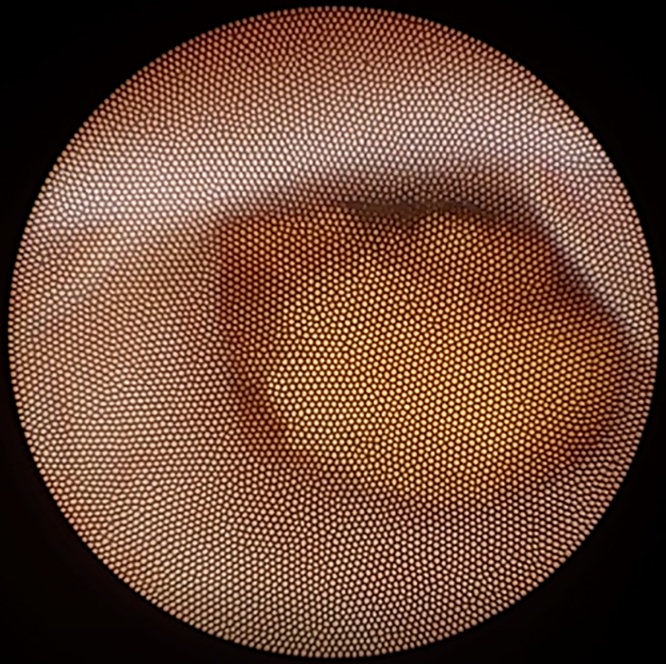
The stone was visualized in the lower part of the ureter.

**Fig. 3:**
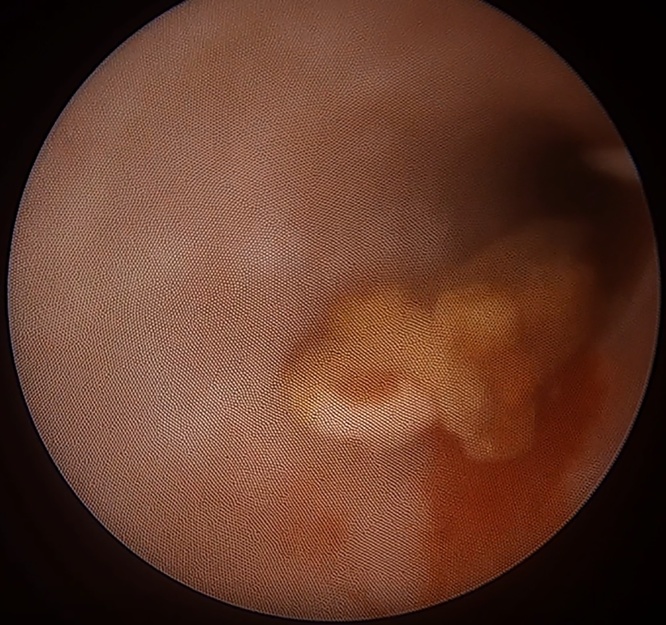
The hem-o-lok clip was identified in the endoscopic view after during the fragmentation of the stone.

**Fig. 4:**
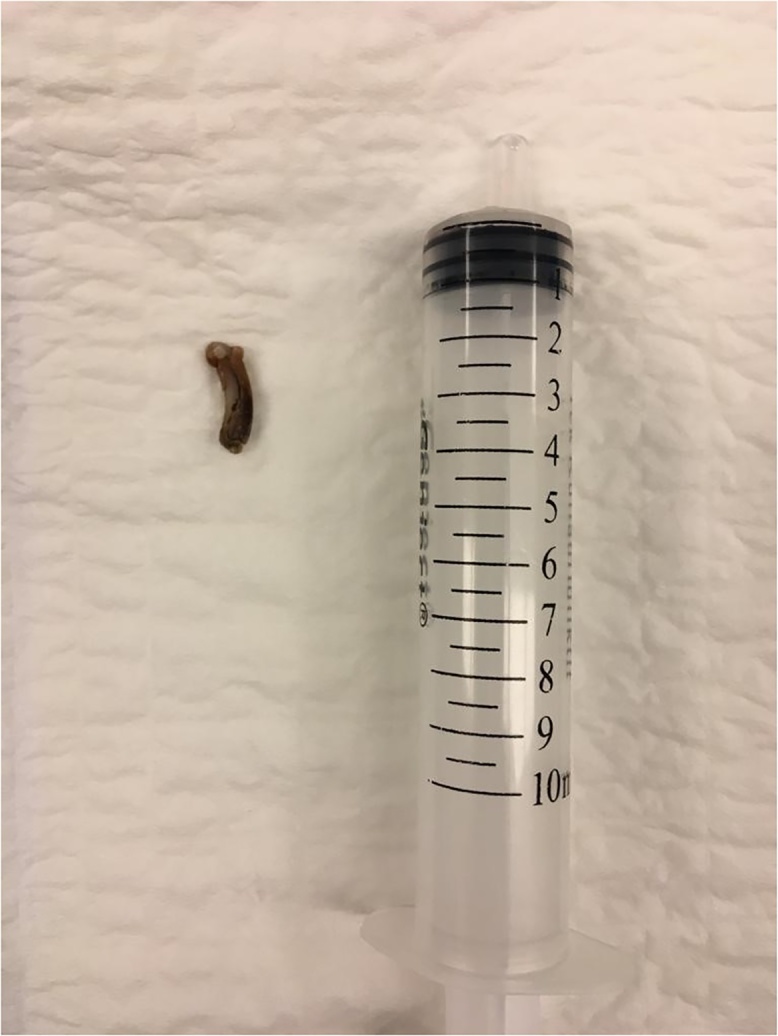
The hem-o-loc clip extracted from ureter with a foreign body forceps.

The patient was placed in modified lithotomy position and an 8 F semi-rigid ureteroscope was introduced through the external urethral meatus. The right ureteral orifice was identified and a 0.035 inch/145 cm safety guidewire was introduced gently into the ureter up to the kidney. A second guide wire was inserted and the railroad technique was used to facilitate the passage of the endoscope along the ureteral lumen. The stone was visualized in the distal ureter (Fig. 2) and fragmentation was initiated using Holmium laser. After disintegrating the cortical rim of the stone, Hem-o-lok clip became visible. Attempts to fragment the clip with the laser failed (Fig. 3) and it was extracted en-bloc using the pincer forceps (Fig. 4). The ureter was inspected fluoroscopically and endoscopically, demonstrating no residual calculus or foreign body. After placing indwelling ureteral and Foley catheters, the operation was completed.

The duration of the procedure was 35 min. The postoperative course was uneventful and the patient was discharged home within 24 h after removing the Foley and indwelling ureteral catheters. At 3-month follow-up, urine and blood work-up were unremarkable and CT scan showed no residual calculus, collecting system dilation, renal mass or metastasis.

## 3 Discussion

Foreign bodies are calculogenic in the urinary tract. Reports have included suture materials, mesh, cotton swabs, metallic clips, embolization coils, and Hem-o-lok clips [[7], [8], [9], [10]]. As minimally-invasive surgical techniques, such as endoscopic PN, become more available, an awareness of these possibilities is prudent [4].

Hem-o-lok clips are standard of care in achieving hemostasis during robot-assisted radical prostatectomy. There are several reports of Hem-o-lock clip migration to the urinary bladder, eventually leading to stone formation [4]. Similar to radical prostatectomy, utilization of endoscopic approaches for partial nephrectomy has increased substantially. In turn, Hem-o-lok clip application for suture anchoring has gained considerable popularity as it speeds up the most time-sensitive part of the surgery [[11], [12], [13]].

Erosion of different type of clips (metal clip, absorbable Lapra-Ty suture clip, Hem-o-lok clip) into the renal collecting system has been documented in the literature [13,14]. Spontaneous discharge of titanium metal clips and Lapra-Ty suture clips without stone formation have been reported [3,[15], [16], [17]]. Park et al. presented a case of ureteral migration of a Hem-o-lok clip after laparoscopic PN and highlighted its successful management with ureteroscopic basket extraction [14]. In another study, Hem-o-lok clip-associated ureteral stone was detected within the context of recurrent urinary tract infection work-up and was then treated with ureteroscopic surgery [3]. “Clip-strasse” after open PN has been described by Bayles et al. who reported retrieval of 3 calcified ureteral hem-o-lok clips by ureteroscopy [12]. Lee et al. reported ureteroscopic management of SWL-resistant renal stone which was formed around the migrated Hem-o-lok clip acting as a core for calculogenesis. [13]]

The mechanism of clip migration into the collecting system following endoscopic PN has not been elucidated yet. Lee et al. claimed that clip migration was related to the unintentional violation of the collecting system during endoscopic PN, which may present as postoperative gross hematuria. Another possible explanation is that suture tension (necessary for optimal parenchymal coaptation during renorrhaphy) may propel the clip to migrate [13]. Regarding our case, he suffered from post-operative hemorrhage and it is possible that clip migration may have been the result of excessive tension along the renal parenchymal suture line, as acknowledged after reviewing the operative report and video recording.

Another controversial issue is the detection of Hem-o-lok clips that have migrated into the collecting system. Variable reports about their CT-visibility exist [18,19]. Matsushita et al have shown that the density of hem-o-lok clip was 223 HU, consistent with its ex-vivo CT-based attenuation measurement [20]. In the current report, the density of the stone was 1609 HU and the stone analysis revealed calcium oxalate monohydrate, which explains the highly dense nature of the aggregate.

In conclusion, clinicians should be aware of the possibility of migrated Hem-o-lok clips serving as a nidus for urinary tract stone formation in patients who have undergone endoscopic PN. Attention to suture tension during endoscopic PN may reduce the risk of clip migration. There is insufficient evidence to support a novel diagnostic workup or treatment plan for clip-associated stones as opposed to routine urinary calculi. Nevertheless, laser lithotripsy of the calculous cortical rim around the Hem-o-lok clip(s) and removal of the denuded foreign body under direct endoscopic visualization are strongly advisable, since Hem-o-lok clips are usually SWL-resistant.

## Informed consent

Written informed consent was obtained from the patient.

## Provenance and peer review

Not commissioned, externally peer-reviewed.

## Conflicts of interest

No conflicts of interest

## Sources of funding

No sources of funding for our research

## Ethical approval

This is not a research study.

## Consent

Written informed consent was obtained from the patient for publication of this case report and accompanying images. A copy of written consent is available for review by the Editor in Chief of this journal on request

## Author contribution

Conceptualization: Murat Can Kiremit, Ersin Koseoglu Methodology: Ersin Koseoglu, Murat Can Kiremit Writing the paper: Murat Can Kiremit, Ersin Koseoglu Investigation: Omer Acar, Mert Kilic Writing- Review- Editing: Yakup Kordan, Mevlana Derya Balbay Visualization: Tarik Esen, Abdullah Erdem Canda Supervision: Tarik Esen, Mevlana Derya Balbay

Project Administration: Yakup Kordan, Omer Acar, Abdullah Erdem Canda Software: Mert Kilic

## Registration of research studies

This is not a research study.

## Guarantor

Murat Can Kiremit Ersin Koseoglu Omer Acar Mert Kilic Yakup Kordan Abdullah Erdem Canda Mevlana Derya Balbay Tarik Esen.
